# Impact of cash transfer programs on healthcare utilization and catastrophic health expenditures in rural Zambia: a cluster randomized controlled trial

**DOI:** 10.3389/frhs.2024.1254195

**Published:** 2024-04-29

**Authors:** Amani Thomas Mori, Mweetwa Mudenda, Bjarne Robberstad, Kjell Arne Johansson, Linda Kampata, Patrick Musonda, Ingvild Sandoy

**Affiliations:** ^1^ Chr. Michelsen Institute, Bergen, Norway; ^2^ Centre for International Health, Department of Global Public Health and Primary Care, University of Bergen, Bergen, Norway; ^3^ Section for Ethics and Health Economics, Department of Global Public Health and Primary Care, University of Bergen, Bergen, Norway; ^4^ Centre for Intervention Science in Maternal and Child Health, University of Bergen, Bergen, Norway; ^5^ Department of Epidemiology and Biostatistics, School of Public Health, University of Zambia, Lusaka, Zambia

**Keywords:** catastrophic health expenditure, healthcare utilization, economic support, cash transfer, cluster-randomized trial, Zambia

## Abstract

**Background:**

Nearly 100 million people are pushed into poverty every year due to catastrophic health expenditures (CHE). We evaluated the impact of cash support programs on healthcare utilization and CHE among households participating in a cluster-randomized controlled trial focusing on adolescent childbearing in rural Zambia.

**Methods and findings:**

The trial recruited adolescent girls from 157 rural schools in 12 districts enrolled in grade 7 in 2016 and consisted of control, economic support, and economic support plus community dialogue arms. Economic support included 3 USD/month for the girls, 35 USD/year for their guardians, and up to 150 USD/year for school fees. Interviews were conducted with 3,870 guardians representing 4,110 girls, 1.5–2 years after the intervention period started. Utilization was defined as visits to formal health facilities, and CHE was health payments exceeding 10% of total household expenditures. The degree of inequality was measured using the Concentration Index. In the control arm, 26.1% of the households utilized inpatient care in the previous year compared to 26.7% in the economic arm (RR = 1.0; 95% CI: 0.9–1.2, *p* = 0.815) and 27.7% in the combined arm (RR = 1.1; 95% CI: 0.9–1.3, *p* = 0.586). Utilization of outpatient care in the previous 4 weeks was 40.7% in the control arm, 41.3% in the economic support (RR = 1.0; 95% CI: 0.8–1.3, *p* = 0.805), and 42.9% in the combined arm (RR = 1.1; 95% CI: 0.8–1.3, *p* = 0.378). About 10.4% of the households in the control arm experienced CHE compared to 11.6% in the economic (RR = 1.1; 95% CI: 0.8–1.5, *p* = 0.468) and 12.1% in the combined arm (RR = 1.1; 95% CI: 0.8–1.5, *p* = 0.468). Utilization of outpatient care and the risk of CHE was relatively higher among the least poor than the poorest households, however, the degree of inequality was relatively smaller in the intervention arms than in the control arm.

**Conclusions:**

Economic support alone and in combination with community dialogue aiming to reduce early childbearing did not appear to have a substantial impact on healthcare utilization and CHE in rural Zambia. However, although cash transfer did not significantly improve healthcare utilization, it reduced the degree of inequality in outpatient healthcare utilization and CHE across wealth groups.

**Trial Registration:**

https://classic.clinicaltrials.gov/ct2/show/NCT02709967, ClinicalTrials.gov, identifier (NCT02709967).

## Introduction

More than one-third of the overall health budgets in many low-income countries are financed through direct out-of-pocket (OOP) payments at health service delivery points (1). In sub-Saharan Africa, it is estimated that 27 out of 48 countries have OOP health payments that exceed 30% of total health expenditure (2). OOP health payments hinder health service utilization (3), and in some situations, the financial burden may be so large that it diminishes a household's capacity to pay for other necessities of life such as food, education, or housing, forcing them into selling properties and assets to cope with the shock (4, 5). Universal Health Coverage (UHC) aims to ensure that every person has access to healthcare without suffering financial hardship. UHC is a high-priority global agenda and is enshrined in the United Nation's resolutions on Global Health and Foreign Policy (6), Universal Health Coverage (7), and Sustainable Development Goals (8).

OOP health payments are usually defined as catastrophic health expenditures (CHE) when they exceed 10% of total household income or 40% of total household non-food expenditures (9, 10). A recent systematic review and meta-analysis study found that the incidence of CHE in sub-Saharan Africa based on the 10% income threshold was 16.5% and 8.7% based on the 40% non-food expenditure threshold (11). Nearly 100 million people are estimated to be pushed into poverty each year as a result of CHE (12). CHE does not always involve large OOP expenditures because even small user fees can be catastrophic to poor families (10, 13). From year 2000 to 2010, the annual incidence of CHE globally increased from 589 to 804 million people, with Africa and Asia being the most affected regions (14).

Zambia is a lower-middle-income country, which spends between 60 and 68 USD per capita on health, of which about 12% is estimated to be from OOP (15, 16). The country relies heavily on donors to finance healthcare, with external health expenditures comprising 43% of current health expenditures. Since her independence in 1964, Zambia has been pursuing different health sector reforms and policies to provide affordable and equitable basic healthcare for all. From independence until 1991, health services were free of charge at the point of use. However, decreasing government financing due to the deteriorating economy led to the introduction of user fees in public facilities in 1992. Exemptions were given to children under the age of five years and people older than 65 years, pregnant women, and those with special conditions such as HIV/AIDS and TB. User fees were abolished in rural health facilities in 2006 and later in urban facilities in 2011 because of the negative impact on health service utilization (17, 18). The establishment of the National Health Insurance Scheme in 2018 was another important milestone for reducing OOP health payments in Zambia (19, 20).

Recent evidence indicates that one in ten households in Zambia experiences CHE in the event of illness (21, 22). Patients are more likely to experience financial hardships if they come from households that are poor, headed by a female, located far from health facilities, or if they visit private health facilities (21). The study by Masiye et al. (2016), which used nationally representative healthcare utilization and expenditure survey data, showed that the likelihood of incurring OOP health payments in Zambia increases with households' income, distance from the health facilities, and increased level of healthcare (23). These factors from Zambia are similar to other sub-Saharan African countries where studies have shown that CHE is associated with poor socioeconomic status, the presence of a person with chronic illness in the household, seeking care from a private provider, living in a rural area, large household size and lack of health insurance among other factors (5, 24).

More than one-third (i.e., 37%) of adolescent girls aged between 15 and 19 years in rural areas in Zambia have started childbearing (25). This is a major concern from a health perspective because early childbearing is associated with an increased risk of complications such as pre-term birth, low birth weight, eclampsia, medical abortion procedures, and post-abortion complications (26, 27). These complications impose a huge economic burden on patients and health systems in Africa (28). Adolescent pregnancy and childbearing also have other undesirable social consequences as they contribute to high school dropout, thus denying young girls their right to education (29, 30). Poverty, high school fees, myths around sexuality and contraceptives, and harmful community norms are some of the contributing factors to adolescent childbearing (31). Therefore, the Research Initiative to Support the Empowerment of Girls (RISE) was a cluster-randomized trial implemented to evaluate the effectiveness of economic support (cash transfers), alone or in combination with community dialogue to reduce adolescent childbearing in rural districts in Zambia (31). The primary outcomes of the trial were the incidence of births and the proportion of girls who sat for the final grade nine exam (and will be published in another paper).

Cash transfers are direct and regular monetary payments provided to poor and vulnerable individuals or households to promote a wide range of social benefits (32, 33). They are classified as conditional if the transfer is tied to the fulfillment of certain obligations, otherwise, they are regarded as unconditional (34). Conditional and unconditional cash transfers are common across the world and often share similar characteristics, however, their designs and implementation strategies are usually adapted to address context-specific challenges. In sub-Saharan Africa unlike in other regions, cash transfer programs tend to focus more on addressing household's immediate needs such as food security and survival, although programs that target behavioral change to reduce prevalence of sexual transmitted infections and early marriage and childbearing are also common (35). In summary, evidence from the systematic review and meta-analysis studies has shown that cash transfer programs can reduce adolescent pregnancy and childbearing (36), poverty, and vulnerabilities (37), as well as improve mental health (38) in Low- and Middle-Income Countries (LMICs).

Cash transfer programs work by increasing the spending capacity of individuals and households, thus overcoming demand-side barriers to social services (39). Lack of resources usually makes poor individuals and households risk-averse (40, 41), which explains the reason cash transfer programs can enhance health service utilization, particularly among poor households (42, 43). In Zambia, evidence from a healthcare utilization and expenditure survey found that a 10% increase in per capita consumption expenditure was associated with a 0.2% increase in OOP health payments (23). Therefore, although economic support in the RISE trial was targeted at reducing adolescent childbearing and improving educational performance, we hypothesized that it could also have an impact on healthcare utilization because enhanced access to healthcare is a priority need in LMICs. It was also hypothesized that a reduction in adolescent pregnancy and the associated costly complications could reduce households' healthcare utilization and exposure to CHE. This study aims to evaluate the overall impact of economic support on healthcare utilization and CHE among the households participating in the RISE cluster-randomized controlled trial in rural Zambia.

## Methods

### Study setting

**Figure 1:**
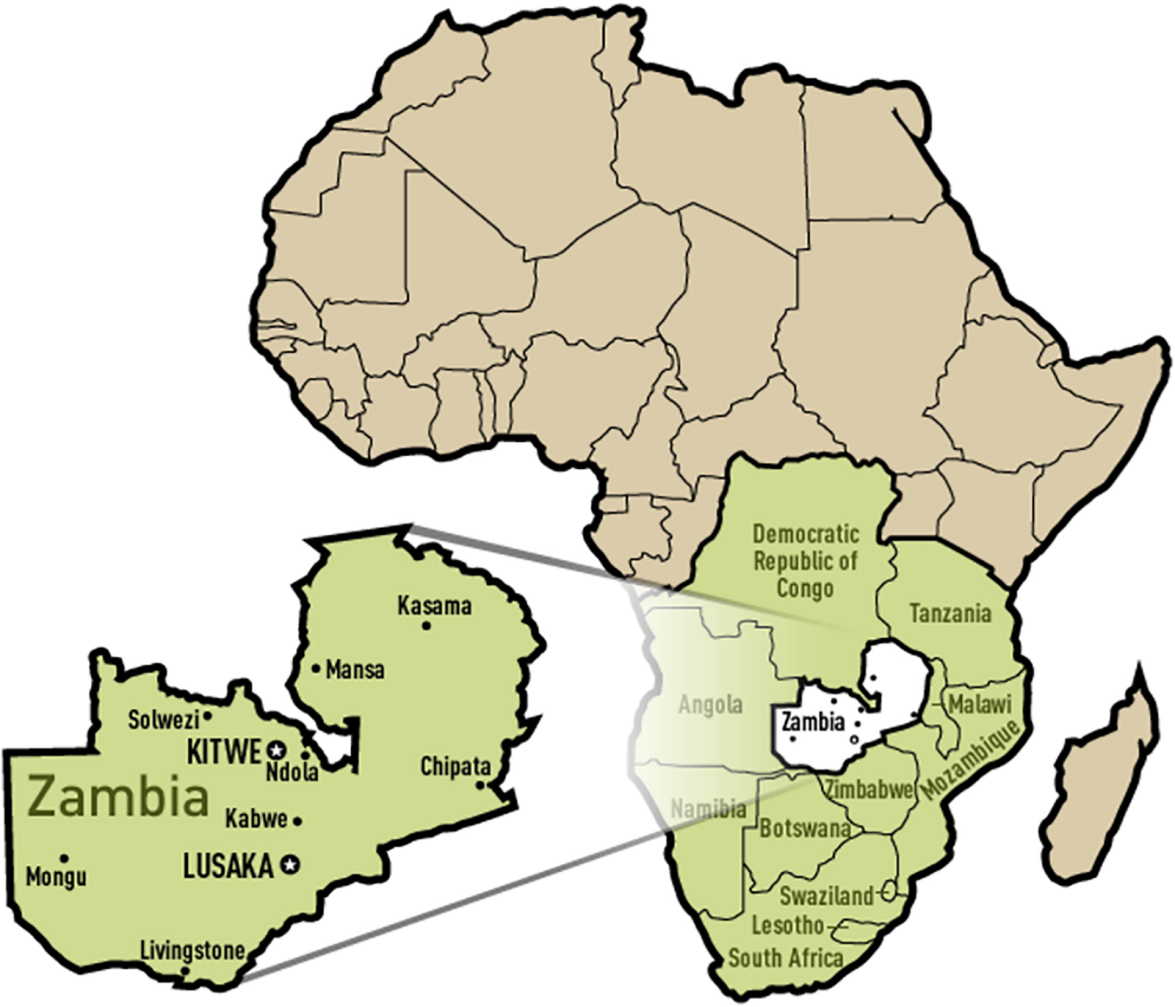
Map of Africa showing the location of Zambia. Source: https://gmlvagabond.blogspot.com/p/zambia_2168.html.

Zambia is located in south-central Africa (Figure 1), and has a per capita gross domestic product (GDP) of 1,316 USD and a population of 17.9 million people as of 2020 (44). About 42% of the population lives in urban areas (45).

### Study design

The household expenditure survey was nested within the RISE Cluster Randomized trial, and the two protocols that detail the effectiveness and economic analyses have been published elsewhere (31, 46). The trial participants were 4,922 girls who were enrolled in 2016 in grade 7 in 157 rural schools (clusters) in the twelve study districts of Kalomo, Choma, Pemba, Monze, Mazabuka, Chikankata, Chisamba, Chibombo, Kabwe, Kapiri Mposhi, Mkushi, and Luano.

The sample size was calculated based on the three primary outcomes i.e., the incidence of births within 8 months of the end of the intervention period, the incidence of births before girls' 18th birthday, and the proportion of girls who sit for the grade 9 exam. We used the 2010 census estimates of the percentages reporting ever giving birth in the study districts to estimate the incidence of childbearing in the control arm: 2% at the average age of 14.5 years, 4% at 15.5 years, 9.5% at 16.5 years, 22% at 17.5 years, and 35% at 18.5 years. The interventions were expected to only affect childbearing at approximately 9 months after the start of the intervention period when the average age would be 15 years. We assumed that 3% of the girls would have given birth before any effects of the interventions could be seen.

We used the Intracluster Correlation Coefficient (ICC) of 0.00737, which was obtained from a similar study conducted in the neighboring country of Malawi (47). The estimated sample sizes of 63 clusters in each intervention arm and 31 in the control arm were found to be sufficient to give >95% power to detect the assumed 40% reduction in the incidence of births before the girls' 18th birthday, and 26.5% increase in the proportion of girls who sit for grade 9 exams between the combined and the control arms. The same sample size would also give 80% power to detect the assumed 25% reduction in the incidence of births before girls' 18th birthday, and a 15% increase in the proportion of girls who sit for grade 9 exams between the economic and the control arms (31).

All the selected schools provided grades 1–9 education, had some mobile telephone coverage, and were at least 8 km apart from each other to minimize the risk of contamination. At least 80% of the eligible participants and their guardians had to assent/consent for their school to be included.

Six randomization ceremonies were organized, each for two districts, where the schools were stratified by district and randomly allocated to the three arms i.e., economic support, combined intervention, and control. Before each ceremony, 1,000 allocations were computer-generated by an independent scientist from the Centre for Interventions Science in Maternal and Child Health (CISMAC), and each allocation was numbered. Officials from the study districts, chiefs, head teachers, members of the Parent-Teacher Association (PTA) of the schools involved in the trial, and members from the community were present. Tickets with numbers corresponding to a specific allocation were drawn from a box. The participants were not blinded. The randomization and the interventions were implemented from September 2016 to November 2018. Below are the descriptions of the arms.

### Economic support arm

In the economic support arm, the girls received monthly cash transfers (CT) of 30 Zambian Kwacha (ZMW) (∼3 USD) while their parents/guardians received 350 ZMW (∼35 USD) annually. The cash support given to parents/guardians was estimated to be sufficient to cover the costs of basic school requirements such as school uniforms and shoes, a school bag, books, and writing materials. In addition, school fees were paid for the girls who managed to advance to junior secondary level i.e., grades 8 and 9. The combination of the annual grant given to the parents/guardians and the payment of school fees essentially made schooling free of cost to the families. The monthly CT for girls was intended to increase the girls' purchasing power instead of relying on boyfriends. Monthly payments were distributed by a committee consisting of a teacher and two parents from the PTA. School fees were paid directly to the school where the girls were enrolled. There was no age limit for receiving economic support for girls who were attending school; however, the support ended after the 18th birthday for girls who dropped out of school.

### Combined economic support plus community dialogue arm

This arm combined economic support with a community-oriented strategy. The latter involved: (1) community and parent meetings promoting supportive social norms around education for girls and delay of early marriage and childbearing; and (2) youth club meetings that were focused on increasing knowledge about sexual and reproductive health. We expected that this strategy would have the added advantage of delaying sexual initiation and increasing the use of modern contraceptives, eventually reducing pregnancy, marriage, and school dropout rates compared to providing economic support alone.

### Control arm

The girls in all the study arms, including the control, were offered some writing materials that included exercise books, pencils, and pens as incentives to encourage them to participate in the study. They were also given an incentive for each of the planned 9 follow-up interviews which they attended as a token gesture to compensate for their time. Apart from this, only standard school and health services were provided to the girls in the control arm.

### Data collection

Baseline interviews were conducted with the parents/guardians of the girls during recruitment in June 2016. Interviews to collect expenditure data were conducted among the parents/guardians of the girls from February 2018 to September 2018, i.e., 1.5–2 years after the intervention period started. Demographic and consumption expenditure data were collected from parents/guardians using a questionnaire administered face-to-face by trained female research assistants. The questionnaire was first prepared in English before it was translated to four local languages (Bemba, Tonga, Lenje, and Nyanja) and back-translated to English again for verification and quality control. The questionnaire was also pre-tested before being used for actual data collection. Data was collected using electronic devices.

**Figure 2:**
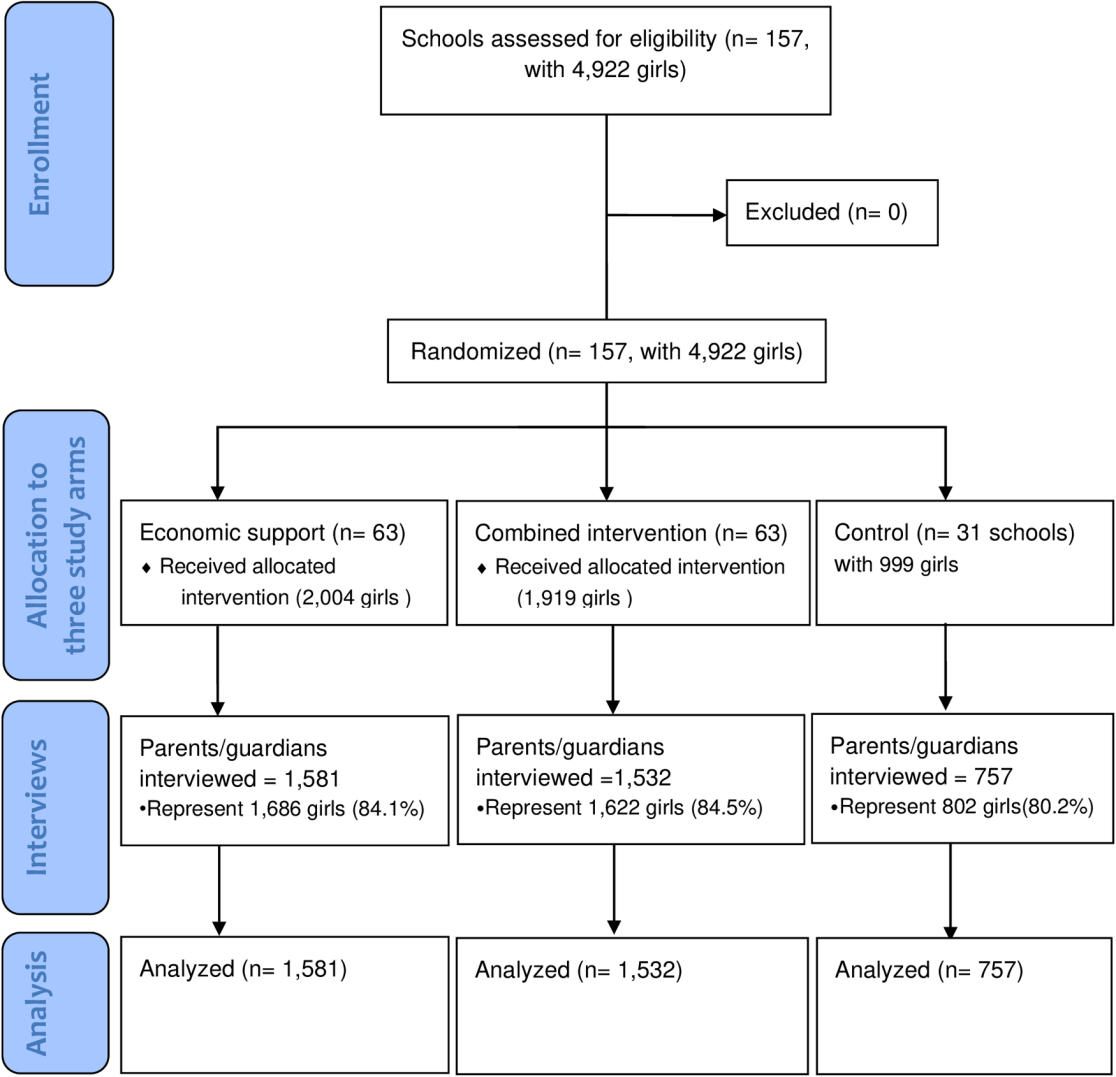
CONSORT flow diagram.

To achieve high participation in the interviews, parents/guardians were invited to meetings at the schools to be informed about the purpose of the planned household expenditure interviews. The invitations were sent through the trial girls themselves and other pupils at the schools (e.g., younger siblings or neighbors). The invitation specified that the preferred person to attend the parent meeting was the guardian who was more knowledgeable about household expenditures to enhance data accuracy. Headteachers and the RISE contact teacher were present at the meetings to help reassure parents. A compensation of ZMW 50 (5 USD at the time) was offered for the time parents spent in the interview. This motivated parents to set aside time to participate in the interview. Research assistants made home visits to those parents/guardians who did not come to the school, and 251 of the parent interviews were held at home. We achieved lower follow-ups for the parent interviews than for the interviews with the participants. This was partly due to families having moved away. For the participants themselves, attempts to interview them over the phone were made if they could not be found in the communities around the included schools, but the parent interviews were unsuitable to do over the phone because they were lengthy. As a result, the household survey was only conducted among 3,870 guardians/parents who attended the parent meetings, representing 4,110 (84%) of the trial participants (Figure 2).

The recall period varied for different categories of items from yearly, monthly, weekly, and daily but were all converted to monthly (expressed per 28 days) expenditures per household.

We used consumption expenditures as a proxy for household welfare. We considered income to be an inappropriate welfare indicator because a large proportion of rural African populations are employed in the informal sector. Therefore, income tends to be highly uneven over time, and in addition, information about it is generally difficult to collect. The questionnaire captured expenditures on a wide range of items consumed by typical rural households in Zambia. Questions were asked about the consumption of food (both purchased and home produce), healthcare, education, and perishable and durable goods. For missing values, we imputed the median expenditure for each item district-wise and by the trial arm to avoid removing any existing price difference between the districts (48). Supplementary Table S1 shows the list of consumption items for which there were missing values. Each of the listed items had approximately 3% missing cost values in all arms.

On food, we asked about the costs of food items bought from the market and the value of home-produced food because most households in rural Africa engage in agriculture and animal husbandry. Aggregate food expenditures per household were capped at 1,500 USD per month to remove unlikely outlier values. This only affected 3 reported outliers.

On education, we asked about tuition fees, examination fees, uniforms, stationery, transport, and costs associated with boarding for each household member who was attending school. We capped aggregate expenditure on education at 2,000 USD per household/month, which affected 4 outliers with reported values above this number.

On health, we asked questions about healthcare seeking among household members who required outpatient or inpatient care. We further asked detailed questions about the direct costs of healthcare, including consultation fees, costs of drugs, supplies and laboratory tests, and cost of transport to and from the health facilities. In addition, we asked about the overall healthcare costs paid at the facility. Whichever was largest, i.e., overall health cost or the sum of itemized costs was used in the final analysis. One observation with very high reported outpatient health expenditures was capped at 2,000 USD per month.

Questions on other expenditures included both perishable and durable items. On perishable items, we asked about routine household expenses on soap, personal care, electricity, petrol/diesel/kerosene, charcoal, batteries, the salary of permanent and seasonal workers, fertilizers and seeds, public transport, and airtime. Under durable goods, we asked about purchase and maintenance costs for cooking utensils, clothes, shoes, radios, cell phones, boreholes, building materials, bicycles, and ox carts. Aggregate expenditures on these items were also capped at 2,000 USD/month, which affected only 2 reported outliers.

### Data analysis

Our unit of analysis was the household. Healthcare utilization was defined as at least one member of a household visiting formal health facilities for outpatient or inpatient health services within the reference period. We measured the proportion of households with CHE using the two definitions that are commonly used in the literature; (i) health expenditure expressed as a proportion of total household expenditure, and (ii) health expenditures as a proportion of non-food expenditures (reflecting capacity to pay) (4, 9, 14, 49, 50). The monthly household health expenditures were considered catastrophic if they exceeded the threshold of 10% of monthly total household expenditures (CHE_T10_) or 40% of the monthly non-food expenditures (CHE_NF40_). We explored the sensitivity of the results by varying the thresholds for total household expenditures (CHE_T_) between 5 and 15% (i.e., CHE_T5_, CHE_T10_, and CHE_T15_) and monthly non-food expenditures (CHE_NF_) between 30 and 50% (i.e., CHE_NF30_, CHE_NF40_, and CHE_NF50_). The degree of inequality in the distribution of CHE_T10_ was measured by the concentration index, which ranges between −1 and 1, with a positive value indicating a higher incidence among the rich and vice versa (9). The further the value of the concentration index is from zero, the higher the degree of inequality within the group.

We calculated household per capita expenditures by using adult equivalent scales, as proposed by Cirto and Michael (51) to be able to compare welfare across households with different sizes and demographic compositions. We used recommended assumptions for poor countries like Zambia and assumed that the consumption cost of a child less than 5 years is 0.3 of that of an adult since expenditures for children tend to be limited, and we used an economies-of-scale factor of 0.9 for adults to reflect a small proportionate cost-saving of being several individuals in the same household since food, which is a private good, typically consumes the largest proportion of household budgets in such settings (48). We then used the per capita expenditures to construct the socioeconomic quintiles. Households were first ranked by decreasing per capita expenditure and then divided into five equal groups. The lowest ranked 20% were categorized as the poorest and the highest 20% as the least poor group.

We plotted Pen's parade graphs to show the distribution of total household expenditures with and without OOP health payments for each arm. The Pen's parade graphs line up every household in ascending order of total expenditures i.e., from the poorest to the least poor on the horizontal axis, while the vertical axis represents the cumulative total household per capita expenditure (9). We used the World Bank's international poverty line of 1.9 USD per day to relate household expenditure to poverty (52).

Impact analysis was done by intention-to-treat with Stata statistical software version 16.1. We used the survey commands to generate means and proportions with 95% confidence intervals, adjusted for clustering. The impact of the interventions on the utilization of healthcare and the proportion of households with CHE was measured by Mixed Effect Generalized Linear Models with log links and binomial distribution, adjusting for the stratified randomization. We used one-way ANOVA to test differences in mean costs for different categories of items. Concentration curves for the intervention arms were plotted against those of the control arm for utilization of outpatient care and inpatient care and CHE. Then dominance tests were conducted using both the Multiple Comparison Approach (MCA) and intersection union principle (IUP) to assess whether the concentration curves for the intervention arms completely lie above that of the control arm as the means to evaluate the impact of the interventions on the distribution of these outcomes. MCA indicates statistical dominance even for one significant difference between the curves in one direction while IUP concludes dominance only if there are significant differences at all points (9). All tests used an alpha level of 5%.

### Ethical considerations

The cluster-randomized trial received ethical clearance from the Biomedical Research Ethics Committee of the University of Zambia (reference number 021-06-15) and the Regional Ethics Committee of Western Norway (Reference number 2015/895). The trial was registered in ClinicalTrials.gov with Reg. No NCT02709967 on 2 March 2016 and in ISRCTN, with Reg. No ISRCTN12727868. on 4 March 2016. The guardians/parents of the girls were asked for written informed consent for the interviews at the time of trial recruitment in 2016. The guardians were also invited to meetings in 2018 immediately before the survey to be oriented about the content and purpose of the household expenditure questions. It was emphasized that the responses they gave to the questions about expenditures would have no consequences for the support they and their daughters were receiving. They were also assured that the information they provided would remain confidential, and the interviews were done in a private location that ensured that others could not listen. The study also received approval from the Ministry of Education to conduct the trial in public schools and to work closely with teachers. It also received permission from chiefs and herdmen to work in their communities.

## Results

### Characteristics of the households

Households that participated in the survey had relatively similar characteristics, except for a slight difference in floor and roofing materials and the use of electricity (Table 1). Chi-square testing did not identify significant differences for these variables.

**Table 1:** Baseline characteristics of the households that participated in the household expenditure survey in 2018.

	Control (*N* = 757)	Economic support (*N* = 1,581)	Combined intervention (*N* = 1,532)
Sex of household head			
Male	38.2	38.3	40.5
Female	1.1	2.0	2.6
Missing	60.7	59.7	56.9
Education (household head)^a^	(%)	(%)	(%)
No formal education	8.7	7.8	10.1
Primary	48.5	47.1	45.5
Junior Secondary	22.0	23.9	22.6
Senior Secondary	14.8	15.1	15.0
Diploma	4.4	4.2	4.9
University	1.6	1.8	2.0
Missing	0.1	0.0	0.0
Occupation (household head)^a^			
Daily wage laborer	3.2	3.9	4.3
Employed	9.1	10.5	13.5
Self-employed in agriculture	62.61	61.9	55.7
Self-employed in business	13.6	12.7	14.8
Not working	1.1	0.8	1.0
Other	2.3	1.8	1.9
# of children <5 years in the household^a^			
0	25.1	26.7	30.6
1	36.3	38.6	35.3
2	20.6	19.9	20.4
3 and above	18.0	13.8	13.6
Missing	-	1.0	0.1
Proportion with health insurance	14.0	12.3	12.5
Floor materials			
Natural/rudimentary floor	52.4	48.8	45.4
Finished floor	47.6	51.2	54.5
Don't know	0.00	0.0	0.1
Roofing materials			
Natural/rudimentary roofing	25.5	28.0	26.1
Finished roof	74.5	72.0	73.9
Other variables			
Households with electricity	14.7	17.9	20.8
Household heads with mobile phones	89.4	90.7	90.4
Households with radio	59.7	59.0	59.5
Households with television	40.2	39.9	43.2
Households with refrigerator	9.0	11.2	11.6
Households with bicycles	67.8	68.4	68.5

^a^ This was not part of the baseline assessment but was asked during the interviews with parents/guardians in the household expenditure survey.

### Expenditures on health, food, education, and other items

The provision of monthly and annual cash support to the girls and their guardians/parents, respectively was associated with a marginal increase in household expenditures for health, food, and other items in the combined arm and food and other items in the economic arm. This led to the total household expenditures being 5% and 3% higher in the combined and economic arms, respectively, compared to the control. Per capita household consumption was 15% higher in the combined arm and 7% higher in the economic than in the control arm (Table 2).

**Table 2:** Consumption expenditure profile (USD per month) of the households.

Parameters	Control *N* = 757		Economic support *N* = 1,581		Combined intervention *N* = 1,532	
Expenditure (USD)	Mean	Median (IQR)	Mean	Median (IQR)	Mean	Median (IQR)
Health	9.2	0 (0–3.9)	7.1	0 (0–4.5)	10.5	0 (0–5.5)
Food	99.7	73.2 (44.3–117.8)	103.7	76.9 (48.3–120.1)	104.2	79.7 (50–125.3)
Education	20.2	8.6 (4.8–16.7)	19.5	7.4 (3.5–15.9)	20.6	7.9 (3.5–17.1)
“Other”	48.8	27.2 (16.3–54.5)	52.7	29.4 (17.4–52.4)	52.0	30.2 (18.0–53.3)
Total	177.9	129.6 (79.8–216.5)	183.0	129.1 (83.1–204.6)	187.2	136.0 (88.8–220.8)
Per capita consumption	34.5	25.6 (14.2–40.0)	36.9	24.7 (15.4–41.2)	39.7	26.5 (16.6–43.5)
Household size	8	7 (6–9)	8	7 (6–9)	8	7 (5–9)

**Figure 3:**
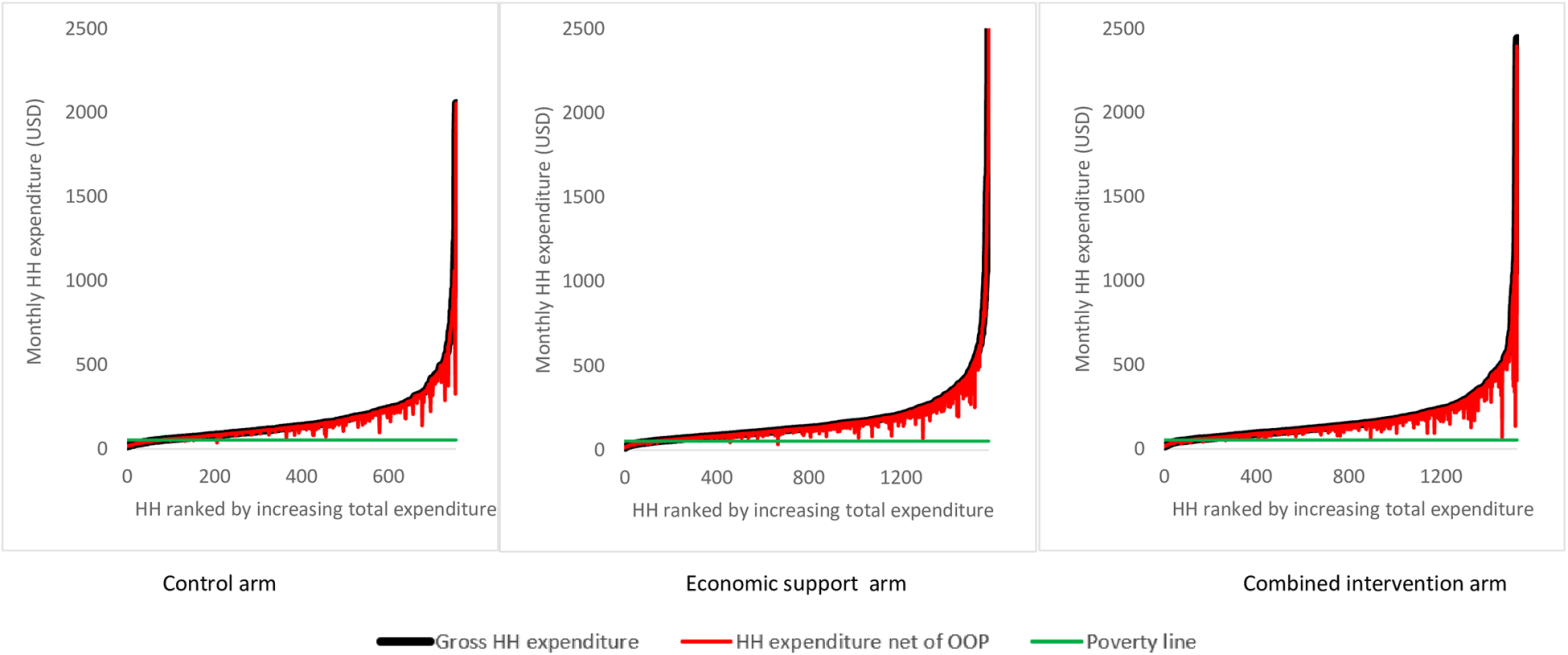
Effect of OOP health payment on Pen’s parade of total household expenditure. HH is an abbreviation for households and OOP is for Out-of-Pocket expenditure. The poverty line cuts the y-axis at i.e., monthly expenditure of 53 USD.

Figure 3 shows Pen's parade graphs, which indicate differences in the total household expenditures in the presence and absence of OOP health payments. In all the arms the OOP health payments contributed a larger share of total household consumption expenditure among the least poor households compared to the poorest households as reflected by the height of the “paint drips”. Further analysis indicated that consumption expenditure in 10.4%, 8.5%, and 7.5% of the households in the control, economic, and combined arms respectively, were below the World Bank's international poverty line of 1.9 USD per day.

### Utilization of healthcare services

**Figure 4:**
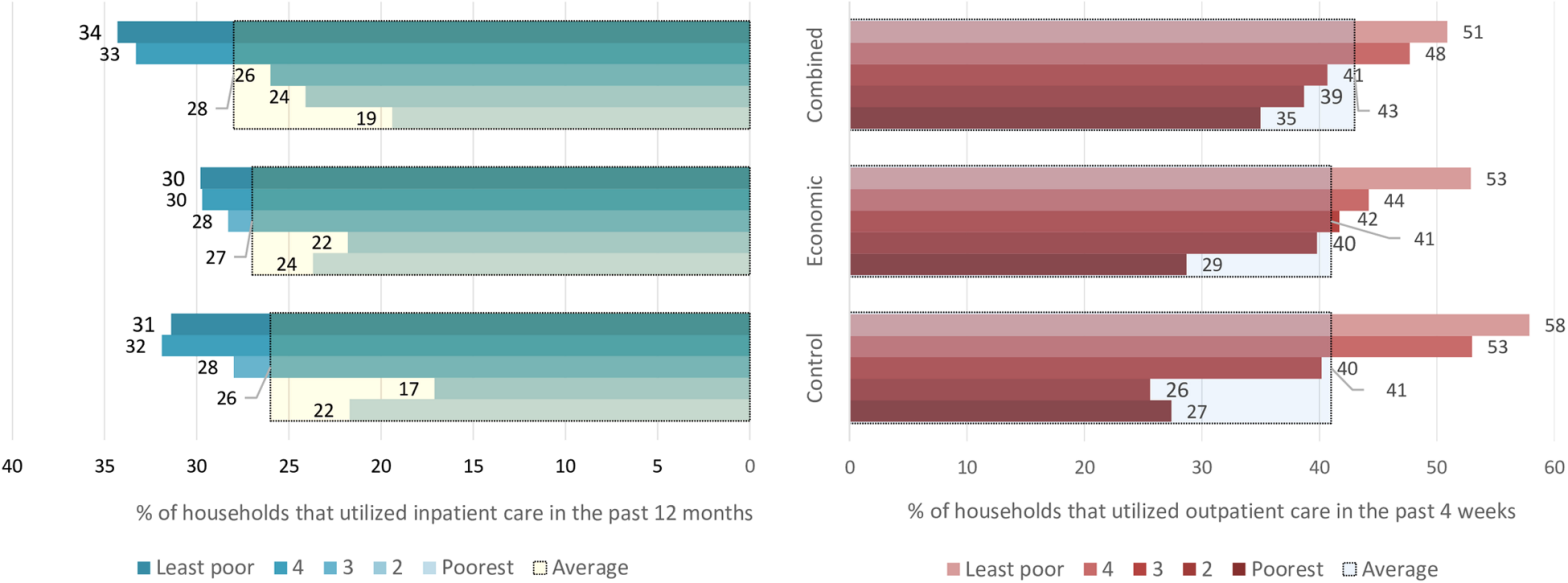
The proportion of households that utilized inpatient and outpatient care. The large bars with black borders indicate overall utilization for each arm, while the small bars indicate utilization for the socioeconomic groups within the arms.

Figure 4 shows the overall utilization of outpatient and inpatient healthcare in the trial arms as disaggregated by socioeconomic status. In the control arm, at least one household member had utilized inpatient healthcare in the previous 12 months in 26% of households, and our analysis indicates no evidence of the interventions having effects on this proportion, i.e., RR = 1.0; (95% CI: 0.9–1.2), *p* = 0.815 for economic support and RR = 1.1; (95% CI: 0.9–1.3), *p* = 0.586 for the combined intervention. Utilization of outpatient healthcare in the previous 4 weeks in the control arm was reported by 41% of households, and the interventions did not appear to affect this proportion either, i.e., RR = 1.0; (95% CI: 0.8–1.3), *p* = 0.805 for economic support and RR = 1.1; (95% CI: 0.8–1.3), *p* = 0.378 for the combined intervention.

When the analysis was disaggregated by socioeconomic status, we still found no clear indication that healthcare utilization was different among households belonging to similar wealth quintiles across the study arms. However, outpatient care utilization among households in the 2nd poorest group was about 55% and 53% higher in relative terms in economic support and combined intervention arms, compared to the households belonging to a similar quintile in the control. Similarly, inpatient care utilization was about 30% and 40% higher in the same group in the economic support and combined intervention arms, respectively compared to the control arm. However, the 95% confidence intervals were relatively wide implying uncertainty around the effect estimates (Table 3). Comparisons between the two intervention groups also did not show an important difference in the proportion of households utilizing healthcare (results not shown).

**Table 3:** Impact of interventions on utilization of healthcare in different socioeconomic groups.

	The relative risk of households reporting using formal care, risk ratio (95 CI)					
	Outpatient care			Inpatient care		
	Control	Economic RR (95% CI)	Combined RR (95% CI)	Control	Economic RR (95% CI)	Combined RR (95% CI)
Poorest	Reference	1.04 (0.75–1.44)	1.24 (0.92–1.68)	Reference	1.06 (0.73–1.55)	0.86 (0.55–1.35)
2	Reference	1.55 (0.98–2.44)	1.53 (0.98–2.40)	Reference	1.28 (0.87–1.87)	1.41 (0.97–2.05)
3	Reference	1.04 (0.77–1.41)	1.01 (0.75–1.38)	Reference	1.02 (0.73–1.42)	0.93 (0.67–1.29)
4	Reference	0.85 (0.67–1.06)	0.93 (0.75–1.15)	Reference	0.94 (0.71–1.24)	1.05 (0.83–1.33)
Least poor	Reference	0.91 (0.73–1.13)	0.89 (0.70–1.13)	Reference	0.95 (0.73–1.23)	1.10 (0.83–1.45)

### Catastrophic health expenditure

**Figure 5:**
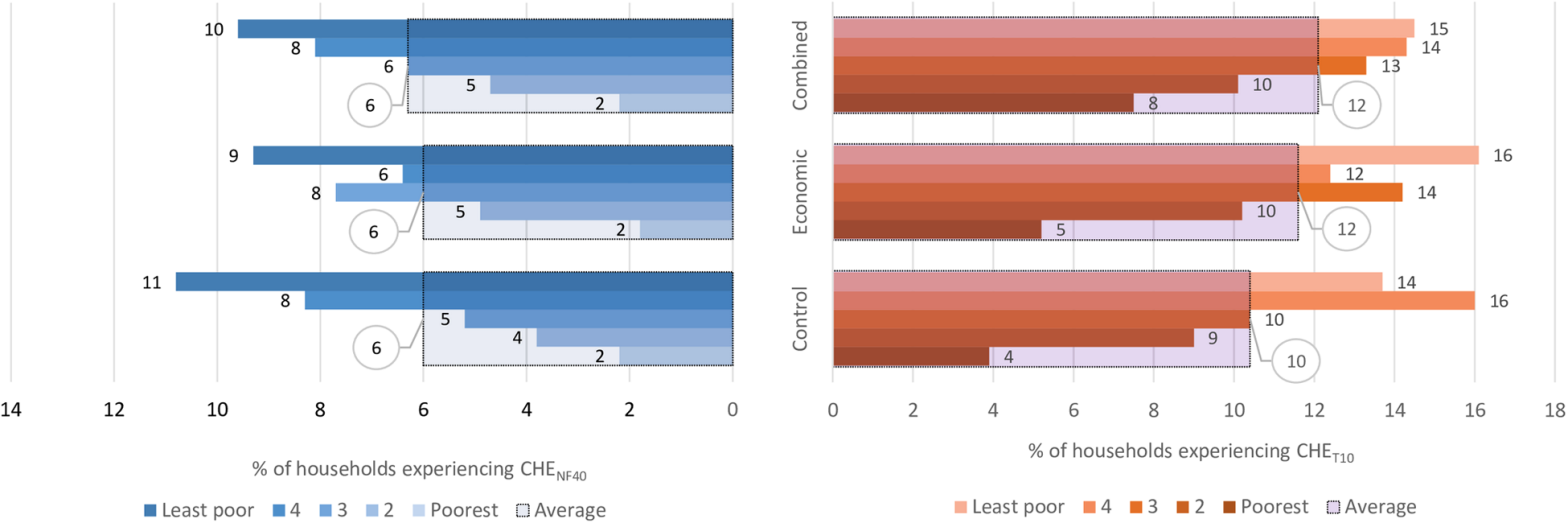
Overall proportion of households with CHET10 and CHENF40 and when disaggregated by socioeconomic status. Key: CHENF40 -Catastrophic health expenditure as 40% non-food expenditures, CHET10-Castrophic health expenditure as 10% of total household expenditure. The large bars with black borders indicate the overall percentage with CHE, while the small bars indicate the percentage with CHE for each socioeconomic group.

Figure 5 shows the overall proportion of households that experienced CHE as a share of total household expenditures (CHE_T10_) and non-food expenditures (CHE_NF40_) and when disaggregated by socioeconomic status. In the control arm, the proportion of households experiencing CHE_T10_ was 10.4%, and the analysis shows that the interventions did not have any clear effects on the incidence of CHE_T10_ with RR = 1.1; (95% CI: 0.8–1.5), *p* = 0.468 for both arms. The proportion of households with CHE_NF40_ in the control arm was 6.0%, and there was also no impact of the interventions on this proportion, i.e., RR = 1.0; (95% CI: 0.7–1.5), *p* = 0.842. Figure 5 also shows that in all the study arms, the least poor households experienced a higher incidence of CHE_T10_ and CHE_NF40_ compared to the poorest households.

Table 4 shows the sensitivity analysis of the impact of the interventions on CHE when expressed as a share of total household expenditures (CHE_T_) and non-food expenditures (CHE_NF_) at different thresholds. When the thresholds were reduced, the proportion of households with CHE increased and vice versa but there was no clear evidence of the interventions producing an impact on CHE at any of the thresholds.

**Table 4:** Impact of the interventions on different thresholds of CHE.

	Percentage of households that reported CHE, % (95% CI)					
	Total household expenditure (CHE_T_) thresholds (%)			Capacity to pay (CHE_NF_) thresholds (%)		
	5%	10%	15%	30%	40%	50%
Control	17.8 (14.1–22.4)	10.4 (7.9–13.7)	7.4 (5.5–9.9)	10.2 (7.9–13.1)	6.0 (4.4–7.9)	4.0 (2.9–5.4)
Economic support	19.0 (16.4–21.8)	11.6 (9.8–13.6)	7.0 (5.7–8.7)	9.5 (7.9–11.4)	6.0 (4.7–7.6)	3.4 (2.6–4.5)
Combined intervention	21.1 (18.4–24.0)	12.1 (10.0–14.5)	7.4 (6.0–9.0)	10.1 (8.3–12.3)	6.3 (5.0–8.0)	3.8 (2.9–5.0)
	Impact of the interventions on the percentage of households that reported CHE, (risk ratio, 95% CI)					
Economic support	1.1 (0.8–1.4)	1.1 (0.8–1.5)	1.0 (0.7–1.4)	0.9 (0.7–1.3)	1.0 (0.7–1.5)	0.9 (0.6–1.3)
Combined intervention	1.2 (0.9–1.5)	1.1 (0.8–1.5)	1.0 (0.7–1.4)	1.0 (0.7–1.3)	1.0 (0.7–1.5)	0.9 (0.6–1.4)

When the impact assessment on CHE was disaggregated by socioeconomic status as shown in Table 5, we found a 90% increased risk of CHE_T10_ in the combined arm and a 30% increase in the economic arm compared to the control in the poorest quintile. However, the 95% CI also includes a 30% and 50% reduced risk, which implies a high degree of uncertainty about the effect size. In the middle group, there was a 40% increased risk of CHE_T10_ in the economic support (*p* = 0.320) and a 30% increased risk in the combined intervention arm (*p* = 0.462) but the 95% confidence intervals also included up to 30% reduced risk. We found similar increase patterns in CHE_NF40_ with economic and combined support for the second and middle quintiles, but these estimates were also highly uncertain.

**Table 5:** Impact of interventions on CHE within socioeconomic groups.

	The relative risk of households reporting CHE, risk ratio (95 CI)					
	10% of total household expenditure (CHE_T10_)			40% of capacity to pay (CHE_NF40_)		
	Control	Economic	Combined	Control	Economic	Combined
Poorest	Reference	1.3 (0.5–3.8)	1.9 (0.7–5.2)	Reference	0.8 (0.2–3.1)	1.0 (0.3–3.7)
2	Reference	1.1 (0.5–2.3)	1.1 (0.5–2.3)	Reference	1.3 (0.5–3.2)	1.2 (0.5–3.4)
3	Reference	1.4 (0.8–2.6)	1.3 (0.7–2.5)	Reference	1.5 (0.8–3.2)	1.3 (0.6–2.7)
4	Reference	0.8 (0.5–1.3)	0.9 (0.5–1.4)	Reference	0.8 (0.4–1.4)	0.9 (0.5–1.6)
Least poor	Reference	1.1 (0.7–1.9)	1.1 (0.6–1.8)	Reference	0.9 (0.5–1.5)	0.9 (0.5–1.6)

### Inequality in healthcare utilization and CHE

**Figure 6:**
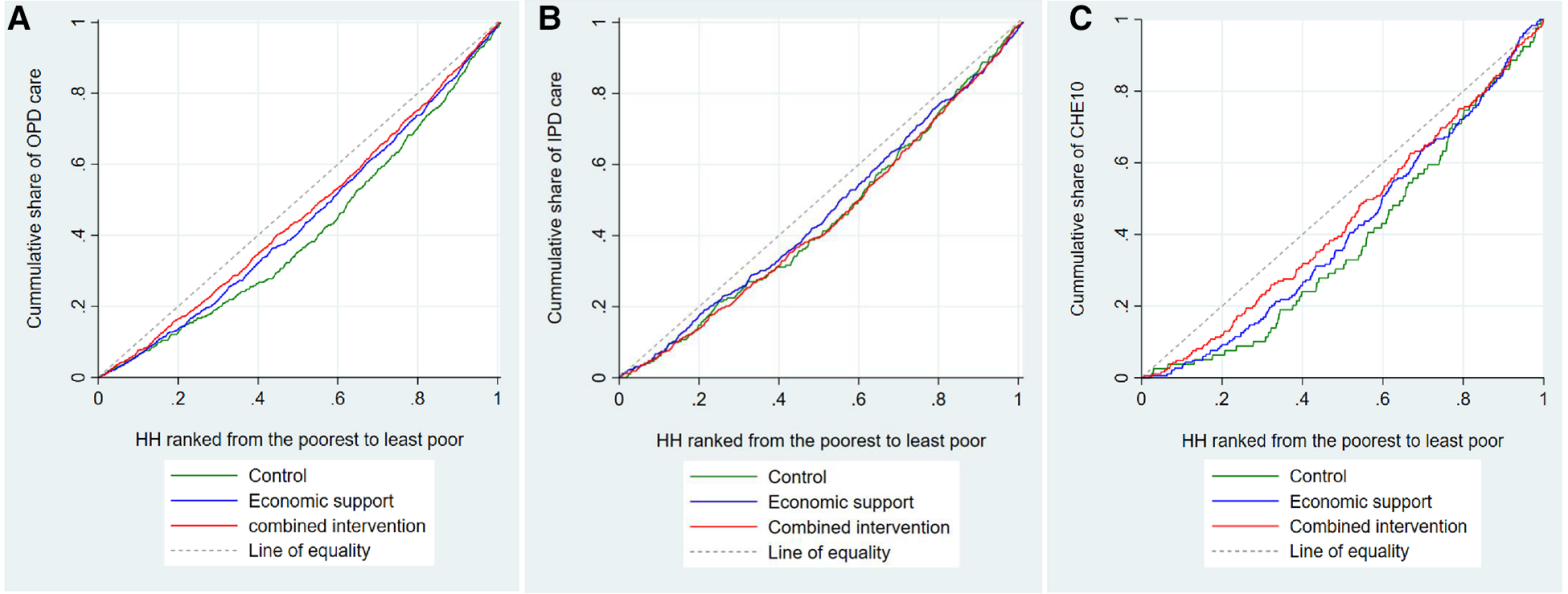
Concentration curves for utilization of outpatient care, inpatient care, and CHE. HH is an abbreviation for household, OPD for outpatient care, IPD for inpatient care, and CHE10 catastrophic health expenditure at 10% of total household expenditure.

In all the trial arms the better-off households utilized outpatient care more than the poorest households. Specifically, the least poor groups in the control, economic, and combined arms utilized outpatient care 200%, 80%, and 40% times more than the poorest groups, respectively (Supplementary Table S2). The concentration indices for utilization of outpatient care in the control, economic support, and combined intervention arms were 0.17, 0.11, and 0.09, respectively. This implies that the degree of inequality in the utilization of outpatient care was relatively less in the intervention arms than in the control arm. Visual inspection of Figure 6A also indicates that the degree of inequality in the utilization of outpatient care was more pro-rich in the control arm than in the intervention arms, although the test of dominance did not completely rule out non-dominance.

As for the utilization of inpatient care, we found that in the combined intervention arm, the three richer quintiles utilized inpatient care more than the poorest households. In the control and economic support arms, we observed similar patterns, but the differences were smaller. The corresponding concentration indices in the control, economic support, and combined intervention arms were 0.10, 0.07, and 0.12, respectively. The test of dominance as well as the visual inspection of Figure 6B, indicates that the degree of inequality in utilization of inpatient care did not differ between the arms.

The risk of the least poor households experiencing CHE_T10_ was almost five times higher than the poorest households in the control arm, three times higher in the economic support arm, and two times higher in the combined intervention arm. The risk of CHE_NF40_ among the least poor households in all three arms was about five times higher compared to the poorest households (Supplementary Table S3). The corresponding concentration index (CI) for CHE_T10_ was 0.21 in the control arm, 0.17 in the economic support arm, and 0.13 in the combined intervention arm. Visual inspection of Figure 6C also indicates that the distribution of CHE was more pro-rich in the control arm than in the intervention arms, although the tests of dominance did not completely rule out non-dominance because the curves overlap towards the upper end.

## Discussion

This study found that 26% and 41% of the households in the control group of this trial in rural Zambia utilized inpatient and outpatient care during the previous four weeks and 12 months, respectively. It further found that about one in ten households in the control group experienced CHE during the previous four weeks. In addition, the provision of economic support in combination with community dialogue slightly increased the overall proportion of households that utilized formal outpatient care (i.e., 40.7% vs. 42.9%) and inpatient care (i.e., 26.1% vs. 27.7%) during the previous four weeks and 12 months, respectively. The incidence of CHE in the previous four weeks also increased slightly from 10.4% to 12.1%. However, there was substantial uncertainty around the effect estimates. We also found that the interventions appeared to have increased the utilization of outpatient healthcare and the incidence of CHE_T10_ among the poorest households, thus reducing the degree of inequality in these outcomes within the arms but there was substantial uncertainty around the estimates.

We believe that the main reason for the lack of clear effect of the interventions on healthcare utilization and CHE could be that the cash support provided to the girls and their guardians was not enough to trigger a large impact on health-seeking behavior and the associated OOP expenditures. There was only a marginal increase in total expenditures for the intervention arms that roughly corresponds to the package of financial support of about 6 USD per month given to the schoolgirls and their guardians. The mean differences were not statistically different but might be considered important from the public health perspective as could imply different purchasing power. This is particularly important among the poorest households as the findings showed a slight improvement in the utilization of outpatient care among the poor and hence a reduced degree of inequality. It is also not certain how much larger cash support would be required to make substantial changes since other studies with higher cash support have also failed to document important effects on healthcare utilization and expenditures. A randomized trial in Tanzania found that a cash transfer ranging between 12 and 36 USD per month (depending on the size of the household) and conditioned on complying with certain health and education conditions, was not associated with a significant increase in expenditures for modern medical care services compared to the control households (53).

Another explanation for the lack of effect on health expenditures could be that health is considered to be a necessary good in the study areas such that an increase in income may not necessarily lead to increased health expenditures (54–56). This contrasts with when health is considered a luxury good, meaning that its use will increase with higher welfare (55). In this study there was an increase in total household expenditures, however, there was only a small increase in mean health expenditures in the combined intervention arm. Economic theory suggests that per-capita health expenditures may increase faster than per-capita increases in income, depending on income elasticities (57).

The incidence of CHE of 10.4% in the control arm is comparable to what has been reported by other studies in rural Zambia (21, 22), but slightly less than the average of 16.5% that was recently reported for sub-Saharan African countries (11). Literature from sub-Saharan Africa (SSA) shows that population-level factors associated with CHE include rural residence, low socioeconomic status, lack of health insurance, large household size, unemployed household head, advanced age (elderly), hospitalization, chronic illness, utilization of specialist healthcare, and utilization of private healthcare providers (24). However, unlike the previous studies, our study which was a cluster randomized trial found that CHE was mostly concentrated among the least poor households than the the poorest in all the study arms. Several studies from Asia (58), Nigeria (59, 60), Egypt (61), and more recently Ethiopia (62) have reported similar findings. Our finding that healthcare utilization was higher among the better-off households is also in line with previous studies from Zambia and Nepal that used data from a large nationally representative household survey (63, 64).

There are several plausible explanations for higher rates of CHE and utilization of formal outpatient and inpatient healthcare among the better-off than among poor families. Evidence from Zambia shows that 20% of the households that used public health facilities ended up incurring OOP (23). In Sri Lanka, a study that used income and expenditure survey data from 42,288 households found that utilization of outpatient and inpatient care was strongly associated with OOP payment under the free healthcare policy, thus imposing a significant burden on the households (65). Therefore, since the poor are the ones who often rely upon public health facilities for affordable services the likelihood of incurring OOP health payments may hinder them from visiting formal health facilities, hence reducing their risk of incurring CHE (66). The risk of OOP health payments may even force poor individuals to delay or forego modern healthcare and revert to self-treatment (67, 68) or seek relatively cheap healthcare from other sources such as herbalists and faith healers. This argument is supported by a study from Nepal, which found that healthcare utilization was pro-rich, and despite services being free in public health facilities, the poor did not utilize them due to high OOP payments (64). For the better-off households, seeking formal healthcare from costly private providers may be associated with high OOP payments, potentially leading to a higher risk of incurring CHE (3, 69). Studies in Zambia have indicated that individuals from better-off families were more likely to seek formal healthcare instead of doing nothing in the event of illness (23, 68).

### Limitations

First, our analysis did not capture all potentially catastrophic effects of illness or disability, such as lost earnings, and was also not able to establish whether the poorest households postponed seeking formal healthcare in the event of illness because they lacked financial resources. Thus, our findings could have underestimated the actual incidence of serious health problems and CHE in the study sample. Second, some consumption items that we enquired about had missing cost information, perhaps due to recall problems. Hence, we could not use multiple imputations since it does not allow the use of items with missing information as independent variables when making imputations to replace missing values. Instead, since cost data were skewed, we used median imputation to replace the missing values. The main disadvantage of this method is that it lowers the variability and disregards relationships between variables. However, since we asked about a long list of consumption items, the results remained robust even when we replaced the median imputed values with zero. This is because only 3% of each of the items had a missing value. Third, although we tried to assure the interviewees that their responses would not affect their cash support, there was a risk that some parents/guardians may still have underreported the household expenses to ensure they did not risk losing the support they were receiving from the project. Fourth, since lack of resources usually makes poor households risk-averse we believe our results are generalizable to other sub-Saharan African countries, where poverty is rampant, particularly among rural communities. We believe we could have seen a substantial impact if cash support was targeted at addressing barriers to accessing healthcare as opposed to education.

## Conclusion

Economic support alone and in combination with community dialogue aiming to reduce early childbearing did not appear to have a substantial impact on healthcare utilization and the incidence of CHE in rural Zambia. Overall, the combination of economic support and community dialogue slightly increased healthcare utilization rates and incidence of CHE but with low precision in effect size. There was also a tendency towards an increased incidence of CHE and utilization of outpatient care among the poorest households, but the precision of the estimates was also low. Therefore, although cash transfer did not significantly improve healthcare utilization, it appears to reduce the degree of inequality in outpatient healthcare utilization and CHE across wealth groups.

## Data Availability

The original contributions presented in the study are included in the article/**Supplementary Material**, further inquiries can be directed to the corresponding author.

## Supplementary material

SUPPLEMENTARY TABLE S1

The proportion of observations with missing values for each item.

SUPPLEMENTARY TABLE S2

Degree of inequality in the utilization of health care. Key: OPD-Outpatient care and IPD-Inpatient care.

SUPPLEMENTARY TABLE S3

CHE by socioeconomic status.
