# Supplementary material for: Impact of cash transfer programs on healthcare utilization and catastrophic health expenditures in rural Zambia: a cluster randomized controlled trial
Source: Front Health Serv. 2024 Apr 29;4:1254195. doi: 10.3389/frhs.2024.1254195 (PMC11089190; doi:10.3389/frhs.2024.1254195)
Supplement: Supplementary file 1 [file Table1.docx]

|  | Control arm  N=757 | | Economic arm  N=1,581 | | Combined arm  N=1,532 | |
| --- | --- | --- | --- | --- | --- | --- |
| Name of item | missing | % | missing | % | missing | % |
| cabbage | 11 | 1.5 | 14 | 0.9 | 14 | 0.9 |
| beans | 34 | 4.5 | 51 | 3.2 | 43 | 2.8 |
| Beef | 13 | 1.7 | 31 | 2.0 | 27 | 1.8 |
| chicken | 10 | 1.3 | 19 | 1.2 | 11 | 0.7 |
| Cooking oil | 29 | 3.8 | 62 | 3.9 | 60 | 3.9 |
| Fish | 11 | 1.5 | 26 | 1.6 | 22 | 1.4 |
| Groundnut | 74 | 9.8 | 113 | 7.1 | 102 | 6.7 |
| Kapenta | 11 | 1.5 | 23 | 1.5 | 22 | 1.4 |
| Maize flour | 40 | 5.3 | 83 | 5.2 | 60 | 3.9 |
| Onions | 12 | 1.6 | 23 | 1.5 | 20 | 1.3 |
| Pork | 1 | 0.1 | 3 | 0.2 | 4 | 0.3 |
| Rape | 20 | 2.6 | 42 | 2.7 | 30 | 2.0 |
| Rice | 13 | 1.7 | 31 | 2.0 | 19 | 1.2 |
| Sugar | 9 | 1.2 | 39 | 2.5 | 41 | 2.7 |
| Tomato | 19 | 2.5 | 29 | 1.8 | 17 | 1.1 |
| Soap | 26 | 3.4 | 45 | 2.8 | 44 | 2.9 |
| Car items | 33 | 4.4 | 51 | 3.2 | 49 | 3.2 |
| Batteries | 21 | 2.8 | 41 | 2.6 | 35 | 2.3 |
| Electricty bills | 11 | 1.5 | 29 | 1.8 | 25 | 1.6 |
| Petrol | 20 | 2.6 | 43 | 2.7 | 36 | 2.3 |
| Charcoal | 15 | 2.0 | 36 | 2.3 | 30 | 2.0 |
| Public transport | 24 | 3.2 | 77 | 4.9 | 77 | 5.0 |
| Air-time voucher | 44 | 5.8 | 80 | 5.1 | 83 | 5.4 |
| Wages employees | 18 | 2.4 | 38 | 2.4 | 28 | 1.8 |
| Seasonal worker | 19 | 2.5 | 51 | 3.2 | 41 | 2.7 |
| Shoes | 27 | 3.6 | 70 | 4.4 | 58 | 3.8 |
| Fertilizer | 26 | 3.4 | 71 | 4.5 | 69 | 4.5 |
| Kitchen utensils | 24 | 3.2 | 58 | 3.7 | 49 | 3.2 |
| Radio | 13 | 1.7 | 33 | 2.1 | 28 | 1.8 |
| Cellphone | 18 | 2.4 | 46 | 2.9 | 40 | 2.6 |
| Oxcart | 19 | 2.5 | 42 | 2.7 | 42 | 2.7 |
| Bicycle | 23 | 3.0 | 52 | 3.3 | 49 | 3.2 |
| Building materials | 26 | 3.4 | 59 | 3.7 | 49 | 3.2 |
| Borehole | 18 | 2.4 | 42 | 2.7 | 38 | 2.5 |
| Cost of opd care | 29 | 3.8 | 41 | 2.6 | 49 | 3.2 |
| Total | **761** |  | **1594** |  | **1411** |  |
| **Average (%)** |  | **3.0** |  | **3.0** |  | **2.7** |
