# Supplementary material for: Impact of cash transfer programs on healthcare utilization and catastrophic health expenditures in rural Zambia: a cluster randomized controlled trial
Source: Front Health Serv. 2024 Apr 29;4:1254195. doi: 10.3389/frhs.2024.1254195 (PMC11089190; doi:10.3389/frhs.2024.1254195)
Supplement: Supplementary file 2 [file Table2.docx]

|  | **Utilization of OPD care within socioeconomic groups Risk Ratio (95% CI)** | | |
| --- | --- | --- | --- |
| Socioeconomic status | Control  RR (95% CI) | Economic support  RR (95% CI) | Combined intervention  RR (95% CI) |
| Poorest | Reference | Reference | Reference |
| 2 | 0.95 (0.59-1.52) | 1.37 (1.10-1.71) | 1.11 (0.91-1.35) |
| 3 | 1.46 (1.04-2.04) | 1.40 (1.09-1.80) | 1.10 (0.90-1.34) |
| 4 | 1.94 (1.38-2.70) | 1.50 (1.20-1.87) | 1.27 (1.07-1.50) |
| Least poor | 2.11 (1.59-2.82) | 1.82 (1.42-2.34) | 1.39 (1.16-1.67) |
|  | **Utilization of IPD care within socioeconomic groups, Risk Ratio (95% CI)** | | |
| Socioeconomic status | Control  RR (95% CI) | Economic support  RR (95% CI) | Combined intervention  RR (95% CI) |
| Poorest | Reference | Reference | Reference |
| 2 | 0.74 (0.48-1.14) | 0.86 (0.67-1.10) | 1.25 (0.87-1.81) |
| 3 | 1.26 (0.88-1.81) | 1.14 (0.85-1.52) | 1.35 (1.06-1.71) |
| 4 | 1.34 (0.99-1.82) | 1.25 (0.95-1.64) | 1.71 (1.22-2.39) |
| Least poor | 1.38 (1.01-1.90) | 1.20 (0.91-1.57) | 1.76 (1.21-2.57) |
