# Supplementary material for: Impact of cash transfer programs on healthcare utilization and catastrophic health expenditures in rural Zambia: a cluster randomized controlled trial
Source: Front Health Serv. 2024 Apr 29;4:1254195. doi: 10.3389/frhs.2024.1254195 (PMC11089190; doi:10.3389/frhs.2024.1254195)
Supplement: Supplementary file 3 [file Table3.docx]

|  | **The relative risk of households reporting CHE, Risk Ratio (95% CI)** | | | | | |
| --- | --- | --- | --- | --- | --- | --- |
|  | **10% of total household expenditure (CHE_T10_)** | | | **40% of capacity to pay (****CHE_NF40_)** | | |
|  | Control  RR (95% CI) | Economic  RR (95% CI) | Combined  RR (95% CI) | Control  RR (95% CI) | Economic  RR (95% CI) | Combined  RR (95% CI) |
| Poorest | Reference | Reference | Reference | Reference | Reference | Reference |
| 2 | 2.5 (0.8-8.5) | 1.9 (1.2-3.2) | 1.4 (0.8-2.4) | 1.7 (0.4-7.6) | 2.7 (1.2-5.9) | 2.1 (0.7-6.3) |
| 3 | 3.1 (1.1-8.9) | 2.7 (1.6-4.6) | 1.8 (1.1-3.1) | 2.4 (0.7-7.9) | 4.3 (1.8-10.1) | 3.0 (1.1-7.9) |
| 4 | 5.3 (2.0-14.0) | 2.4 (1.4-4.0) | 2.0 (1.2-3.2) | 3.9 (1.2-12.3) | 3.5 (1.5-8.3) | 4.0 (1.7-9.6) |
| Least poor | 4.5 (1.7-11.7) | 3.1 (1.6-5.9) | 2.0 (1.2-3.3) | 5.1 (1.9-14.0) | 5.2 (2.0-13.2) | 4.7 (1.8-12.3) |
